# Siamese neural network-enhanced electrocardiography can re-identify anonymized healthcare data

**DOI:** 10.1093/ehjdh/ztaf011

**Published:** 2025-02-25

**Authors:** Krzysztof Macierzanka, Arunashis Sau, Konstantinos Patlatzoglou, Libor Pastika, Ewa Sieliwonczyk, Mehak Gurnani, Nicholas S Peters, Jonathan W Waks, Daniel B Kramer, Fu Siong Ng

**Affiliations:** National Heart and Lung Institute, Imperial College London, Hammersmith Campus, Du Cane Road, London W12 0NN, UK; National Heart and Lung Institute, Imperial College London, Hammersmith Campus, Du Cane Road, London W12 0NN, UK; Department of Cardiology, Hammersmith Hospital, Imperial College Healthcare NHS Trust, Du Cane Road, London W12 0NN, UK; National Heart and Lung Institute, Imperial College London, Hammersmith Campus, Du Cane Road, London W12 0NN, UK; National Heart and Lung Institute, Imperial College London, Hammersmith Campus, Du Cane Road, London W12 0NN, UK; National Heart and Lung Institute, Imperial College London, Hammersmith Campus, Du Cane Road, London W12 0NN, UK; MRC Laboratory of Medical Sciences, Imperial College London, London, UK; Faculty of Medicine, University of Antwerp and Antwerp University Hospital, Antwerp, Belgium; National Heart and Lung Institute, Imperial College London, Hammersmith Campus, Du Cane Road, London W12 0NN, UK; National Heart and Lung Institute, Imperial College London, Hammersmith Campus, Du Cane Road, London W12 0NN, UK; Department of Cardiology, Hammersmith Hospital, Imperial College Healthcare NHS Trust, Du Cane Road, London W12 0NN, UK; Harvard-Thorndike Electrophysiology Institute, Beth Israel Deaconess Medical Center, Harvard Medical School, Boston, MA, USA; National Heart and Lung Institute, Imperial College London, Hammersmith Campus, Du Cane Road, London W12 0NN, UK; Richard A. and Susan F. Smith Center for Outcomes Research in Cardiology, Beth Israel Deaconess Medical Center, Harvard Medical School, Boston, MA, USA; National Heart and Lung Institute, Imperial College London, Hammersmith Campus, Du Cane Road, London W12 0NN, UK; Department of Cardiology, Hammersmith Hospital, Imperial College Healthcare NHS Trust, Du Cane Road, London W12 0NN, UK; Department of Cardiology, Chelsea and Westminster Hospital NHS Foundation Trust, 369 Fulham Road, London SW10 9NH, UK

**Keywords:** Artificial intelligence, Electrocardiogram, Siamese neural network, Identification, Continuous monitoring

## Abstract

**Aims:**

Many research databases with anonymized patient data contain electrocardiograms (ECGs) from which traditional identifiers have been removed. We evaluated the ability of artificial intelligence (AI) methods to determine the similarity between ECGs and assessed whether they have the potential to be misused to re-identify individuals from anonymized datasets.

**Methods and results:**

We utilized a convolutional Siamese neural network (SNN) architecture, which derives a Euclidean distance similarity metric between two input ECGs. A secondary care dataset of 864 283 ECGs (72 455 subjects) was used. Siamese neural network-electrocardiogram (SNN-ECG) achieves an accuracy of 91.68% when classifying between 2 689 124 same-subject pairs and 2 689 124 different-subject pairs. This performance increases to 93.61% and 95.97% in outpatient and normal ECG subsets. In a simulated ‘motivated intruder’ test, SNN-ECG can identify individuals from large datasets. In datasets of 100, 1000, 10 000, and 20 000 ECGs, where only one ECG is also from the reference individual, it achieves success rates of 79.2%, 62.6%, 45.0%, and 40.0%, respectively. If this was random, the success would be 1%, 0.1%, 0.01%, and 0.005%, respectively. Additional basic information, like subject sex or age-range, enhances performance further. We also found that, on the subject level, ECG pair similarity is clinically relevant; greater ECG dissimilarity associates with all-cause mortality [hazard ratio, 1.22 (1.21–1.23), *P* < 0.0001] and is additive to an AI-ECG model trained for mortality prediction.

**Conclusion:**

Anonymized ECGs retain information that may facilitate subject re-identification, raising privacy and data protection concerns. However, SNN-ECG models also have positive uses and can enhance risk prediction of cardiovascular disease.

## Graphical Abstract

**Graphical Abstract:**
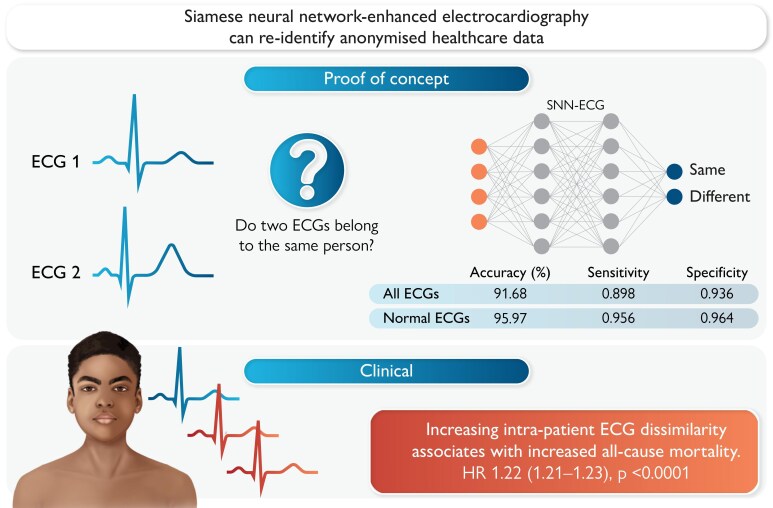

## Introduction

Many large datasets of anonymized patient or volunteer data are available for research.^1,2^ Data access is granted to researchers on the assumption that individuals cannot be identified from these anonymized data. Indeed, assurance of ‘de-identification’ underpins a broad regulatory, legal, and bioethical framework for conducting research with these anonymized individual-level data. However, concerns have been raised regarding the potential to re-identify subjects in large datasets,^3^ for example, using genomic data.^4,5^ While genetic data can be expected to be unique and identifiable, a more common data source, the electrocardiogram (ECG), is also known to carry features that are unique to individuals.^6^ This raises the possibility of using the ECG as a biometric test to identify specific individuals from anonymized datasets.

Artificial intelligence (AI) has already demonstrated utility within healthcare and has the potential for further, significant benefit.^7^ Deep learning, applied to the ECG, has been particularly revolutionary, with diagnostic and predictive models surpassing human performance for a wide range of tasks.^8–11^ However, although AI-ECG models can clearly benefit patient care, there is the possibility that they can be misused for unethical purposes.

A specific deep learning architecture, the Siamese neural network (SNN), is commonly used on image or sound data for the purposes of individual authentication by estimating the similarity of a pair of inputs. Briefly, the SNN architecture is based on identically-weighted, parallel encoder networks which output embeddings of input data. The distance between these embeddings approximates their similarity. Siamese neural network approaches have been applied in healthcare, not only for the diagnosis of disease^12,13^ but also by using output similarity measures to serve as continuous estimates of disease severity and progression.^14^

We developed a Siamese neural network-electrocardiogram (SNN-ECG) model for two purposes. Firstly, we aimed to test whether these models have the potential to be misused by a ‘motivated intruder’^15^ to re-identify individuals from anonymized healthcare data, and thus highlight potential ethical and data protection issues surrounding such AI models. Secondly, we tested the clinical utility of the SNN-ECG model and hypothesized that increasing dissimilarity within a subject’s ECGs may indicate clinical deterioration and could therefore be useful for clinical risk prediction.

## Methods

### Ethical approvals

This study complies with all relevant ethical regulations; further details are provided in the Supplementary material online, *Methods*.

### Electrocardiogram pre-processing

Twelve-lead ECGs were pre-processed with a bandpass filter 0.5–100 Hz, a notch filter at 60 Hz, and re-sampling to 400 Hz (more details in Supplementary material online, *Methods*). As leads III, aVL, aVR, and aVF are linear combinations of leads I and II, these leads were not used for model development or evaluation. Single median beats were created from each 10 s ECG using the BRAVEHEART ECG analysis software as previously described.^16^

### BIDMC cohort

We conducted our study using the secondary care Beth Israel Deaconess Medical Center (BIDMC) cohort, comprising routinely collected data from Boston, USA. We only included subjects with between 4 and 75 ECGs each (see Supplementary material online, *Figure S1*). The inclusion of subjects with larger numbers of ECGs was explored but did not improve model performance whilst increasing model training time. Data were initially split 60:10:30% at the subject level into training, validation, and hold-out test sets to prevent data leakage. We developed our model using triplets that comprise three ECGs—an anchor ECG from a subject (A), another ECG from the same subject (positive, P), and an ECG from a different subject (negative, N). All possible triplets were generated within each set by first creating all unique anchor positive (AP) pairs per subject. The negative ECG was a random ECG from a random other subject. After filtering for hard triplets,^17^ the final subject split became ∼50:10:40% (details in Supplementary material online, *Methods*).

### Siamese neural network model development

**Figure 1:**
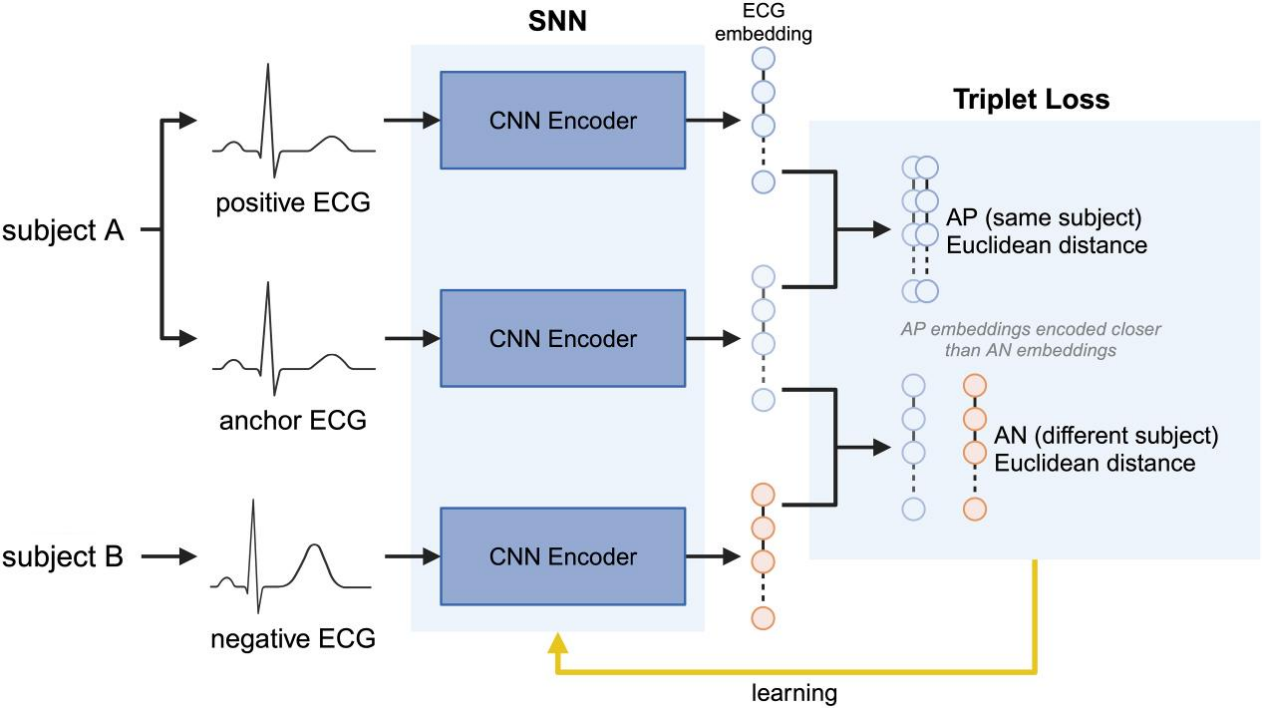
The SNN architecture and triplet loss function: the SNN receives three ECG inputs: a reference ECG from a given subject A (anchor), another ECG from the same subject A (positive), and an ECG from a different subject B (negative) and encodes embeddings for these using three identical CNNs. The Euclidean distances between anchor and positive embeddings and between anchor and negative embeddings are calculated, with the triplet loss function updating the weights of the CNN to encode anchor-positive embeddings closer together than anchor-negative embeddings. AN, anchor-negative; AP, anchor-positive; CNN, convolutional neural network; SNN, Siamese neural network.

We employed a convolutional SNN architecture with a triplet loss function^17^ trained on subject identity labels (*Figure 1*). The model receives three ECG inputs (a triplet), whereby a positive ECG and negative ECG are evaluated against an anchor ECG to allow the model to learn what characterizes ECG pairs from the same (AP) and from different (anchor negative, AN) subjects. An embedding vector is computed from each input with the use of a one-dimensional convolutional neural network (CNN) encoder; the architecture and weights of the encoder are identical for each input (see Supplementary material online, *Methods* for details). The final convolutional layer is flattened to create a 128-node embedding from which Euclidean distances are calculated. The triplet loss function minimizes the Euclidean distance between AP embeddings and maximizes the Euclidean distance between AN embeddings (see Supplementary material online, *Figure S2*). We employed hard triplets to aid model convergence (see Supplementary material online, *Methods* for details).^17^ The model iteration with the lowest validation loss was used for testing.

### Model evaluation

The Euclidean distance for all AP and AN pairs in the validation set was computed. The intersection of the AP and AN Euclidean distance distributions in the validation set was used as a binary threshold for analyses in the hold-out test set (see Supplementary material online, *Table S1*). For analyses conducted in outpatient, normal, left bundle branch block (LBBB), right bundle branch block (RBBB), and atrial fibrillation (AF) subsets, ECGs matching these criteria (described below) from the validation and hold-out test sets were filtered. All possible triplets were created for each subset (validation and hold-out test data did not mix). A binary threshold was computed as above for each subset. We trained a separate model, SNN-ECG-Normal, using only the normal ECG subset (see Supplementary material online, *Methods*, Supplementary material online, *Table S2*).

The model was developed and tested using the Keras^18^ API for Tensorflow^19^ in Python 3.10.12. Statistical analysis was conducted in the R statistical environment.

### Data subsets

Normal ECGs were determined by searching for ‘normal ecg’ in the cardiologist free text reports, a whole word match was required in order to exclude ‘abnormal ecg’. Electrocardiograms with the phrase ‘otherwise’ were also excluded from the normal definition. Additionally, we filtered by heart rate (60–100 b.p.m.), PR interval (<200 ms), QRS duration (<120 ms), and QTc interval (<470 ms).^10^

A previously described CNN classifier was used to define the LBBB, RBBB, and AF diagnoses on 10 s 12-lead ECGs.^20^ No fine-tuning of this model was performed. Manual verification of model predictions was performed in small subset of ECGs. The performance of this diagnostic approach has been shown to be superior to medical professionals.^20^

### Re-identification task

**Figure 2:**
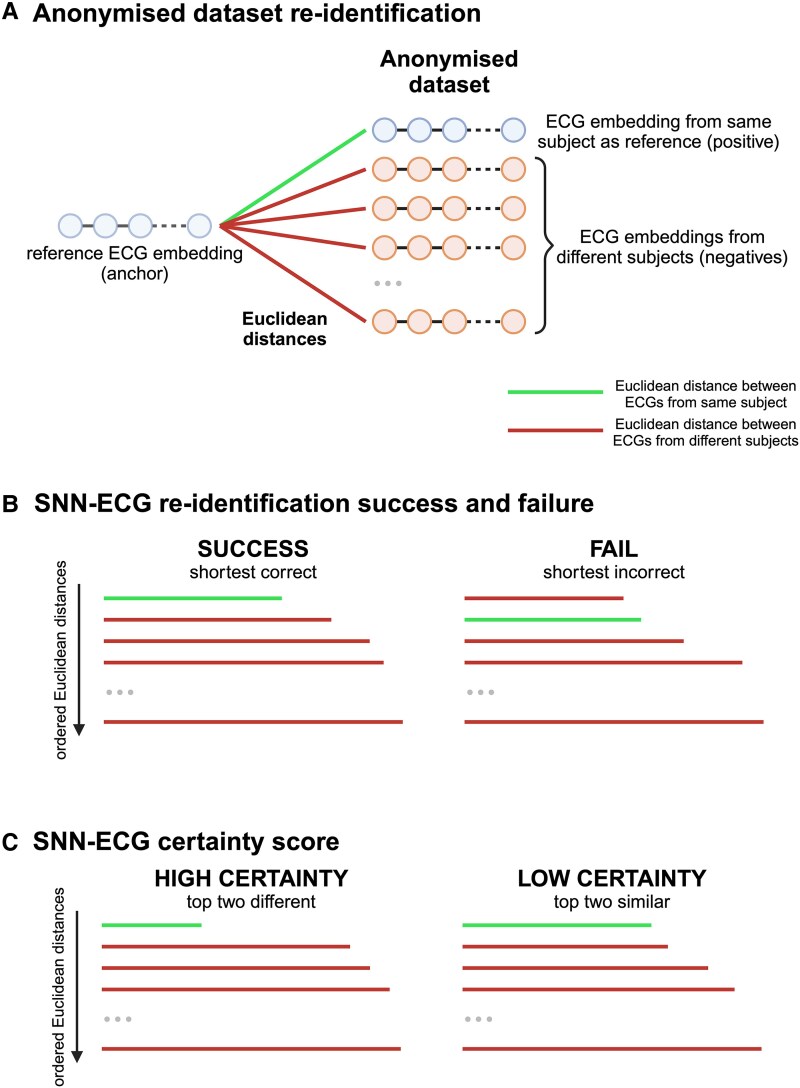
Re-identification of a subject from an anonymized dataset: (*A*) A visualization of the re-identification process during which Euclidean distances are calculated between a subject’s reference ECG and all other ECGs in an anonymized dataset (where only one also belongs to that subject). The Euclidean distance for the only correct ECG pair (i.e. that belonging to the same subject) is shown in green, and the Euclidean distances for all other incorrect ECG pairs is shown in red. (*B*) SNN-ECG is only successful at re-identification when the Euclidean distance between the two ECGs belonging to the reference subject is the shortest. (*C*) In a real-world setting, where the correct ECG from the anonymized dataset is not known, a certainty score is output alongside the model’s top choice to aid interpretation. As the discrepancy between top two ECGs from the anonymized dataset that are closest to the reference ECG increases (i.e. their Euclidean distances are most different), the model is more confident of its top choice. SNN, Siamese neural network.

We simulated the ‘motivated intruder’ test^15^ to evaluate the ability of the model to use a reference subject’s ECG to identify them from a group where only one ECG also belongs to that subject, by creating anonymized datasets of 100, 1000, 10 000, or 20 000 ECGs; in these, only one ECG belonged to the reference subject. A Euclidean distance was calculated between the reference ECG and all ECGs in the dataset (*Figure 2A*), and the model correctly identified the subject if the Euclidean distance between the two ECGs belonging to the reference subject was the shortest (*Figure 2B*). We conducted this analysis for all test subjects (for all dataset sizes), and all subjects within each subset (for datasets sizes of 100 and 1000 ECGs), with at least 2 ECGs in the respective (sub)set—this was therefore the number of trials (see Supplementary material online, *Table S3*). These repeat trials eliminated the variability of results intrinsically linked to the random selection of a subject’s reference ECG and other ECG in the dataset when a subject had more than two ECGs from which to choose. It also eliminated the variability attached to the selection of the 99 or 999 other random ECGs for the dataset. Reported result represents the proportion of trials in which the re-identification task was successful, out of all trials.

We also conducted this analysis in datasets filtered for the reference subject’s decade age bracket, their sex, and both, at the time of acquisition of the ECG to assess model performance when supplemented with demographic information often attached to anonymized records.

To better simulate a real-world scenario where the correct subject within the dataset is not known, we generated a certainty score of model choice for the datasets of 100 and 1000 ECGs (*Figure 2C*). Once all 100 or 1000 Euclidean distances had been computed, the discrepancy between the two shortest Euclidean distances was calculated by subtracting one from the other. The model is less confident when this discrepancy is small. In the validation dataset, the discrepancy above which 95% of trials had the correct model choice was selected as the threshold for 95% certainty. This threshold was applied to the test datasets to identify trials where the 95% certainty score threshold was met.

### Per-subject temporal analysis

To determine if ECG similarity is associated with patient outcome, we generated all AP pairs for each individual with multiple ECGs in our hold-out test set. Per-subject accuracy was defined as the number of their AP pairs correctly classified by SNN-ECG. The per-subject average accuracy rate is weighted by the *n* ECGs per subject. We also assessed this on the subject level for chronologically successive ECG pairs, ECGs paired with only the subject’s first ever ECG, and ECGs paired with only the subject’s last ever ECG. We also filtered for AP pairs comprising just outpatient ECGs and analysed model performance when placing minimum time constraints between chronologically successive outpatient ECGs.

### Comparison with artificial intelligence-electrocardiogram model for mortality prediction

We recently described AIRE (the AI-ECG Risk Estimation platform), which is an AI-ECG model trained with a supervised machine learning approach to predict *time-to-mortality*.^10^ We created Cox models to evaluate the additive value of SNN-ECG Euclidean distance to AIRE model predictions in predicting all-cause mortality. In order to approximately fix the time duration between ECGs, subjects with ECGs around 6 months apart (between 150 and 210 days apart) were selected. A Cox model was fit with age, sex, AIRE predictions for both ECGs and SNN-ECG standardized Euclidean distance.

## Results

### Siamese neural network-electrocardiogram accurately identifies electrocardiograms from same or different subjects

The SNN-ECG model was developed using the BIDMC dataset,^10^ comprising 72 455 secondary care subjects with between 4 and 75 ECGs each, totalling 864 283 ECGs.

**Figure 3:**
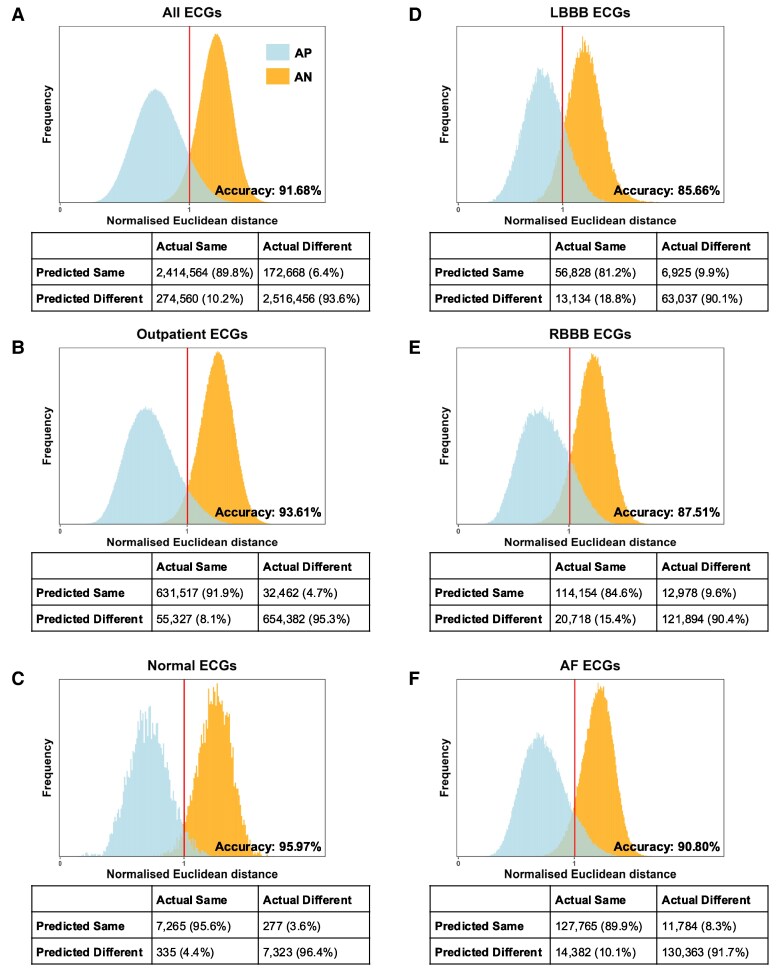
Distributions of AP and AN pair normalized Euclidean distances: these are shown for (*A*) all hold-out test subjects (*n* subjects = 21 737) and subsets of (*B*) outpatient (*n* = 17 085), (*C*) normal (*n* = 3438), (*D*) LBBB (*n* = 1438), (*E*) RBBB (*n* = 2139), and (*F*) AF (*n* = 4206) subjects. The binary thresholds, overall accuracies, and confusion matrices are shown. If there was perfect discrimination between AP and AN pairs, there would be no overlap between the two histograms. AF, atrial fibrillation; AN, anchor-negative; AP, anchor-positive; LBBB, left bundle branch block; RBBB, right bundle branch block.

The distributions of AP and AN normalized Euclidean distances for all test triplets are shown in *Figure 3*. The normalized Euclidean distance is defined as the Euclidean distance divided by the binary threshold value. Relative to the binary threshold, SNN-ECG achieves an accuracy of 91.68%, a sensitivity of 0.898, a specificity of 0.936, a positive predictive value (PPV) of 0.933, and a negative predictive value (NPV) of 0.902 for 2 689 124 triplets in the hold-out test set. We found SNN-ECG performs better in ECGs without obvious abnormalities with accuracies of 93.61% and 95.97% in outpatient and normal ECGs, respectively, and 85.66%, 87.51%, and 90.80% in LBBB, RBBB, and AF, respectively (*Table 1*, Supplementary material online, *Table S1*). The performance of SNN-ECG-Normal is overall decreased relative to SNN-ECG (see Supplementary material online, *Table S2*).

**Table 1:** Siamese neural network-electrocardiogram results for the hold-out test set and electrocardiogram subsets

	Total triplets	Accuracy (%)	Sensitivity	Specificity	PPV	NPV
All	2 689 124	91.68	0.898	0.936	0.933	0.902
Outpatient	686 844	93.61	0.919	0.953	0.951	0.922
Normal	7600	95.97	0.956	0.964	0.963	0.956
LBBB	69 962	85.66	0.812	0.901	0.891	0.828
RBBB	134 872	87.51	0.847	0.904	0.898	0.855
AF	142 147	90.80	0.899	0.917	0.916	0.901

AF, atrial fibrillation; LBBB, left bundle branch block; NPV, negative predictive value; PPV, positive predictive value; RBBB, right bundle branch block; SNN, Siamese neural network.

### Factors affecting Siamese neural network-electrocardiogram performance

In order to further investigate the factors that influence SNN-ECG performance, we evaluated how time between ECG recordings for AP ECG pairs (i.e. from the same subject) affected the Euclidean distance returned by the model. We found a direct relationship between increasing time between AP ECG acquisitions and their Euclidean distance (see Supplementary material online, *Figure S3*). AP pairs comprising both outpatient ECGs also had a higher rate (91.94%) of being correctly classified as from the same subject than AP pairs comprising both inpatient ECGs (88.24%) or an outpatient and inpatient ECG (85.51%).

### Siamese neural network-electrocardiogram identifies subjects from anonymized datasets

In our simulated ‘motivated intruder’ test,^15^ using a single reference ECG from a known individual, SNN-ECG can identify that individual from an anonymized dataset. The model achieves success rates of 79.2%, 62.6%, 45.0%, and 40.0% for datasets of 100, 1000, 10 000, and 20 000 ECGs, respectively. If this was random, the success would have been 1%, 0.1%, 0.01%, and 0.005%, respectively. For the subset of normal ECGs, SNN-ECG achieves success rates of 89.4% and 72.5% for datasets of 100 and 1000 ECGs, respectively. This improves when additional basic information is supplied to the model. Success rates of 91.4% and 75.9% occur when supplemented with subject sex, 95.1% and 84.2% with subject decade age bracket, and 96.3% and 87.5% with both, for datasets of 100 and 1000 normal ECGs, respectively. Results for all hold-out test subjects, as well as outpatient, LBBB, RBBB, and AF subsets are also shown in *Table 2*. For datasets of 10 000 and 20 000 subjects, the experiments were not conducted for the ECG subsets due to insufficient ECGs available for each subset. The success rates for the correct subject being identified in the top 2, 5, and 10 SNN-ECG choices are shown in Supplementary material online, *Table S4*. There is ∼6–8% point increase in success rates comparing those for the first and top two returned subjects. This is consistent across subsets and dataset sizes.

**Table 2:** Siamese neural network-electrocardiogram success rates for subject re-identification from anonymized datasets

	Dataset size	No supplemental information (%)	Sex (%)	Decade age bracket (%)	Sex and decade age bracket (%)
All	**100**	79.2	82.8	88.9	91.4
	**1000**	62.6	66.4	74.7	78.4
	**10 000**	45.0	48.2	56.9	60.6
	**20 000**	40.0	43.1	51.3	54.7
Outpatient	**100**	85.3	87.7	92.2	94.0
	**1000**	70.8	73.8	80.6	83.4
Normal	**100**	89.4	91.4	95.1	96.3
	**1000**	72.5	75.9	84.2	87.5
LBBB	**100**	64.6	70.1	77.7	82.5
	**1000**	44.6	49.1	57.7	62.6
RBBB	**100**	66.7	71.9	77.8	81.6
	**1000**	51.9	55.2	62.5	66.3
AF	**100**	73.0	77.2	83.7	87.7
	**1000**	54.2	58.6	67.2	71.0

If choice was random, the success would be 1%, 0.1%, 0.01%, and 0.005% in datasets of sizes 100, 1000, 10 000, and 20 000, respectively.

AF, atrial fibrillation; LBBB, left bundle branch block; RBBB, right bundle branch block; SNN, Siamese neural network.

### Siamese neural network-electrocardiogram certainty score allows estimate of model confidence

Siamese neural network-electrocardiogram consistently achieves 97–100% accuracy when the certainty score is at the 95% level or greater, for all test subjects as well as the outpatient, normal, LBBB, RBBB, and AF subsets in datasets of both 100 and 1000 subjects (*Table 3*, Supplementary material online, *Table S5*). Siamese neural network-electrocardiogram achieves this certainty score in 10 693/21 737 (49.2%) and 6278/21 737 (28.9%) trials for all test subjects, and in 997/1539 (64.8%) and 507/1539 (32.9%) trials for the normal subset in datasets of 100 and 1000 subjects, respectively.

**Table 3:** Proportions of datasets with shortest Euclidean distance identifying one available anchor positive pair, stratified by model certainty

Dataset size	100		1000	
	Datasets with certainty > 95% (%) (of *n* datasets)	Datasets with certainty ≤ 95% (%) (of *n* datasets)	Datasets with certainty > 95% (%) (of *n* datasets)	Datasets with certainty ≤ 95% (%) (of *n* datasets)
All	98.8 (of 10 693)	60.3 (of 11 044)	98.7 (of 6278)	48.0 (of 15 459)
Outpatient	99.3 (of 8551)	64.7 (of 5814)	98.9 (of 5211)	54.8 (of 9154)
Normal	99.3 (of 997)	71.2 (of 542)	99.6 (of 507)	59.2 (of 1032)
LBBB	99.2 (of 257)	54.5 (of 884)	98.4 (of 246)^a^	38.3 (of 895)^a^
RBBB	97.8 (of 672)	47.8 (of 1105)	97.5 (of 476)^b^	38.3 (of 1301)^b^
AF	99.5 (of 998)	60.5 (of 2095)	100.0 (of 129)	52.2 (of 2964)

AF, atrial fibrillation; LBBB, left bundle branch block; RBBB, right bundle branch block.

^a^Only 516 LBBB subjects in the validation set hence hold-out test dataset size was limited to 516.

^b^Only 674 RBBB subjects in the validation set hence hold-out test dataset size was limited to 674.

### Siamese neural network-electrocardiogram for continuous monitoring

**Figure 4:**
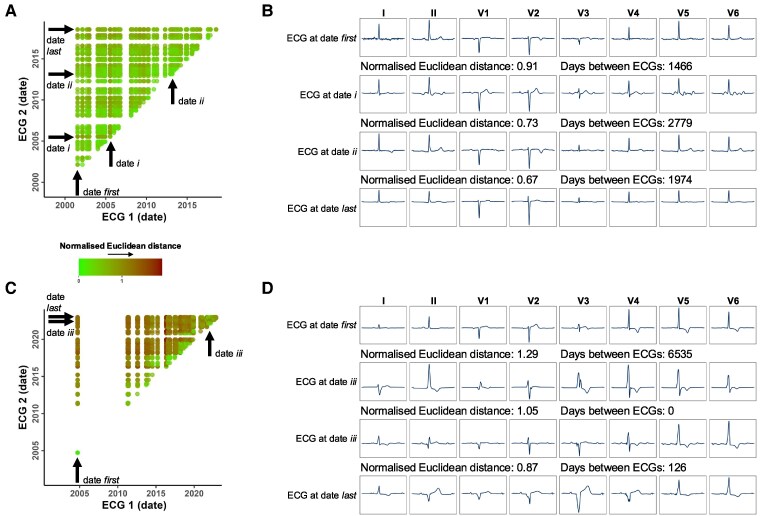
Evolution of ECG similarity over time: data for two subjects with multiple ECGs, from the hold-out test set, are shown. (*A*) and (*B*) are from one subject, and (*C*) and (*D*) are from a second subject. (*A*) and (*C*) show the normalized Euclidean distances for all ECG pairs. A point represents a unique ECG pair and is coloured by normalized Euclidean distance as output by SNN-ECG with smaller distances in green and larger distances in brown. The chronologically successive ECG pairs are plotted along the bottom-left to top-right diagonal. The normalized Euclidean distance generally increases towards the top-left corner with increasing time between the acquisition of ECGs in a given AP pair. (*B*) and (*D*) show four ECG median beat traces for each subject. In (*B*), these correspond to the first recorded ECG, subsequent ECGs from dates *i* and *ii*, and the last recorded ECG. In (*D*), these correspond to the first recorded ECG, a subsequent ECG from date *iii*, a further ECG also from date *iii*, and the last recorded ECG. The normalized Euclidean distance and temporal difference (in days) between the four ECGs is shown. A normalized Euclidean distance < 1 indicates that the model correctly predicts these two ECGs to be from the same subject. Regardless of the time between ECGs, the model can account for slight variations within a subject’s ECGs, but begins to fail when ECG traces show gross changes in morphology, as in (*D*) with evolving QRS morphology, even on the same day.

In order to determine how SNN-ECG performs on the subject level, we analysed all AP pairs for each subject from the hold-out test set (two subjects are shown in *Figure 4A* and *C*). We found an overall accuracy of 96.98% when considering all the chronologically successive ECGs of all the test subjects. We also analysed this for ECGs paired with only the subject’s first ever ECG or last ever ECG, where the overall accuracy was 86.16% and 88.98%, respectively. When filtering for AP pairs comprising just outpatient ECGs, the overall accuracy was 97.11% for chronologically successive outpatient ECGs and 88.24% when a subject’s outpatient ECGs were only paired with their first recorded outpatient ECG. The overall accuracy was 96.78% and 95.66% for successive outpatient ECGs at least 1 month and at least 1 year apart, respectively. This suggests that ‘failure’ of SNN-ECG to identify correct ECG pairings is likely to represent genuine changes in the ECG and/or clinical condition of the patient.

The visualization of select ECG traces for subjects (*Figure 4B* and *D*) shows that the model is able to account for slight variations within a subject’s ECGs, even after prolonged periods of time between ECGs. Siamese neural network-electrocardiogram begins to fail when a subject’s ECG traces begin to manifest gross changes.

### Siamese neural network-electrocardiogram predicts deterioration

Based on the findings above, we hypothesized the evolution of Euclidean distances output by SNN-ECG over time is associated with the patient’s clinical trajectory. In order to standardize the temporal difference between ECGs, we only evaluated ECG pairs between 150 and 210 days apart. From the hold-out test set, 98 175 ECG pairs from 9385 subjects met this criterion. In a Cox model adjusted for age and sex, standardized distance between ECG pairs was associated with a hazard ratio of 1.22 (1.21–1.23, *P* < 0.0001) for risk of all-cause mortality. In cases where both ECGs were taken from outpatient encounters, the hazard ratio reduced to 1.13 (1.10–1.16, *P* < 0.0001). We also investigated the additional value of SNN-ECG compared to our recently described mortality prediction model (the AI-ECG Risk Estimation platform, AIRE).^10^ Standardized distance was additive to AIRE predicted mortality applied to each ECG (adjusted HR 1.15 (1.14–1.17), *P* < 0.0001).

## Discussion

We report an SNN-based deep learning model for subject identification, trained on subject identity rather than ECG diagnosis. Our model is 91.68% accurate in triplet analyses, when differentiating a pair of ECGs from the same individual versus a pair of ECGs from different individuals. We are the first to test the SNN in a ‘motivated intruder’ test and first to report the likelihood of subject re-identification from large, anonymized datasets, of different sizes. The SNN can identify a known individual’s ECG from a dataset of 1000 normal ECGs, with a success rate of 87.5%. Although we do not aim to re-identify individuals from anonymized datasets, we want our results to highlight the potential of misuse of AI-ECG models, to inform data protection practices. Apart from the ‘motivated intruder’ test, another key novelty of our work is our demonstration of the potential clinical utility of SNN-ECG models to predict the risk of adverse outcomes in patients.

### Siamese neural network-electrocardiogram performs better for normal electrocardiograms

Although previous reports on subject identification from ECGs exist,^21–23^ ours is the first utilizing a considerably larger, clinical dataset. The BIDMC dataset, with which our model was trained and tested, is comprised of normal and abnormal ECGs. Importantly, the superior performance of SNN-ECG relative to SNN-ECG-Normal implies the model looks past the disease when exposed to it during training, only learning what links same-subjects and what distinguishes different-subjects. The model performs better in normal ECGs than in those where pathology is present. This worse performance in abnormal ECGs is most likely not explained by the manner in which abnormal ECGs subsets were constructed, but by the pathological ECG waveform itself, which may obscure signatures relevant to individual identification. Interestingly, for ECGs with obvious abnormalities, SNN-ECG performs better in subsets of AF than LBBB or RBBB. There may be pathophysiological reasons behind this, for example AF ECGs may retain a more subject-specific QRS morphology, which is not the case in LBBB or RBBB where ECGs morphologies could be more ‘generic’. Alternatively, LBBB and RBBB QRS morphology may be more sensitive to electrode placement thus generating less similar ECGs for a given subject.

### Re-identifying subjects from anonymized datasets

We demonstrated that SNN-ECG can re-identify subjects from anonymized datasets, raising potential ethical and legal concerns. Considering a hypothetical scenario whereby a researcher has access to an ECG from a known individual and that individual is also a volunteer in a large research database, the researcher could potentially apply an SNN-ECG model to all the ECGs in the anonymized research database, and re-identify that individual, thus gaining access to that individual’s sensitive linked data, such as medical history and genetic data. Knowing the individual’s sex and age bracket aids this task. We show that anonymized datasets of up to ∼1000 individuals are susceptible to such problems, with success rates of 96.3% and 87.5% in datasets of 100 and 1000 normal ECGs, respectively. These datasets may, therefore, not pass the ‘motivated intruder’ test.^15^ As expected, model performance decreases for larger datasets, but even in a dataset of 20 000 ECGs, the model still has a 40.0% success rate in identifying the single correct ECG, without any supplemental information. The current level of accuracy may not be an imminent threat for large research databases, but is a factor that needs to be considered for smaller research databases.

### Continuous similarity scores in artificial intelligence models

Additionally, we have demonstrated how the continuous nature of the Euclidean distance can be output into a certainty score reflecting model confidence of subject choice from an anonymized dataset. We found higher certainty choices corresponded to significantly higher success rates. On the other hand, when the model chose incorrectly, this was reflected in lower levels of certainty. Integration of confidence estimates such as these in AI models can provide helpful context.

### Continuous similarity scores for risk estimation

Analogous to previous approaches in other domains,^24^ we have demonstrated for the first time that intra-subject ECG dissimilarity associates with increased risk of all-cause mortality. Although dissimilarity could imply clinical improvement or decline, our findings suggest that in general decline is more likely. Our model can be supplemented with existing AI-ECG models that predict mortality and, importantly, is additive to AIRE, our model trained using supervised machine learning, for mortality prediction.^10^

Used clinically, the ECG trace lends itself to being classified into discrete categories of disease or health, without analysis along a continuous severity metric between these two states. This decreases the precision of the ECG as an investigative and diagnostic tool. An SNN-based approach exploits the non-discrete nature of the ECG to provide similarity on a continuous spectrum. In the present study, this is the spectrum of subject identity, but can reasonably be finetuned to estimate pathology severity change, as has been the case in retinopathy of prematurity,^14^ osteoarthritis,^14^ diabetic foot disease,^25^ and cancers,^26–28^ which use non-ECG image inputs. Analogously, directional SNN approaches also provide metrics of treatment response through the analysis of longitudinal computed tomography or magnetic resonance imaging data.^29,30^ Our findings provide an additional mechanism for precision healthcare, continuing the trend away from the potentially artificial tendency to cluster disease into discrete categories of severity and aetiology.

### Limitations

The reduced numbers of ECGs demonstrating an abnormality (AF, RBBB, LBBB) mean that an exact conclusion about SNN-ECG performance per pathology cannot be made. This is especially true for results pertaining to our model identifying a subject from datasets of LBBB or RBBB ECGs where our group sizes were limited by the number of patients in the validation set. The present work is also limited by the lack of external validation and model interpretation, such as saliency mapping or variational autoencoder-based explanation approaches. Finally, it would be worthwhile to assess the performance of subject identification, and especially clinical deterioration, in a model similar to ours, but with only a single ECG lead input. This would be more reflective of ambulatory data, for example from smart watches, and can be the subject of future work.

## Conclusion

Siamese neural network-electrocardiogram models can be applied for clinical benefit but also have the potential to be misused to circumvent data protection measures. These models can be deployed to re-identify a subject using only their 12-lead ECG, and thus ECGs, especially in small research datasets, should be considered identifiable data, with additional data protection measures being required. On the positive side, SNN-ECG can also be used to monitor changes in clinical condition, with ECG dissimilarity over time being associated with increased clinical risk. While SNN-ECG presents a potential concern for privacy and data protection in anonymized research datasets, it simultaneously offers an additional tool for risk prediction and precision medicine.

## Supplementary Material

ztaf011_Supplementary_Data

## Data Availability

The BIDMC dataset is restricted due to ethical limitations. The underlying code for this study is not publicly available but may be made available to qualified researchers on reasonable request to the corresponding author.
