# Supplementary material for: Siamese neural network-enhanced electrocardiography can re-identify anonymized healthcare data
Source: Eur Heart J Digit Health. 2025 Feb 25;6(3):417–26. doi: 10.1093/ehjdh/ztaf011 (PMC12088719; doi:10.1093/ehjdh/ztaf011)
Supplement: ztaf011_Supplementary_Data [file ztaf011_supplementary_data.docx]

**Siamese neural network-enhanced electrocardiography can re-identify anonymised healthcare data**

**Supplementary Appendix**

**Table of Contents**

[Supplementary Methods 3](#_Toc183152519)

[Supplementary Figure 1 5](#_Toc183152520)

[Supplementary Figure 2 6](#_Toc183152521)

[Supplementary Figure 3 7](#_Toc183152522)

[Supplementary Table 1 8](#_Toc183152523)

[Supplementary Table 2 8](#_Toc183152524)

[Supplementary Table 3 9](#_Toc183152525)

[Supplementary Table 4 10](#_Toc183152526)

[Supplementary Table 5 12](#_Toc183152527)

[References to Supplementary Appendix 13](#_Toc183152528)

# Supplementary Methods

**Ethical approvals**

For the Beth Israel Deaconess Medical Center (BIDMC) cohort, ethics review and approval was provided by the Beth Israel Deaconess Medical Center Committee on Clinical Investigations, IRB protocol #2023P000042.

**ECG pre-processing**

ECGs originally sampled at 500Hz were down-sampled to 400Hz. This has no effect on performance but improves training times. (1,2)

**SNN model development – CNN architecture and model hyperparameters**

The one-dimensional convolutional neural network (CNN) encoder consists of 8 sequential blocks. Each block consists of a one-dimensional convolution layer, followed by batch normalization and a LeakyReLU activation. The convolutional filter count increases across blocks: starting with 8 filters in the first two blocks, then progressing through 16, 32, 64, 128, 512, and finally 1024 filters in the last block. Max pooling layers follow the 16-, 64-, and 512-filter blocks, and dropout is applied in most blocks with rates ranging from 0.2 to 0.35 to prevent overfitting. The final block is flattened to create a 128-node embedding. ReLU activation is used here. The choice of the architecture was informed by initial experiments to make the network sufficiently deep to extract pertinent information.

Training uses a triplet loss function with a margin of 1500. The margin hyperparameter was increased sequentially in initial trials to maximise performance. We use the Adam optimiser and a learning rate of 1×10^−5^, over 50 (SNN-ECG) or 100 (SNN-ECG-Normal) epochs (**Supplementary Figure 2**) with a batch size of 32.

**Hard triplets**

Hard triplets are employed in SNNs to force the model to learn truly distinguishing features. (3) *A priori* Euclidean distances between initially created AP and AN pairs in the training set were computed using ECG embeddings created as part of an unsupervised, feature extraction method. At this stage, AP pairs deemed too similar (~35%), and AN pairs deemed too dissimilar (~45%), were permutated to avoid this, thus creating hard triplets. This did not decrease the number of train ECGs but did decrease the number of train triplets. The final training, validation, hold-out test split became ~50:10:40%.

**SNN-ECG-Normal**

To develop the SNN-ECG-Normal model, we filtered for normal ECGs in the original train, validation and hold-out test subjects. There were 22,732 ECGs from 11,760 subjects that met these criteria. These ECGs were used to make hard triplets as before. We evaluated the performance of SNN-ECG-Normal in all the ECG subsets, including the whole hold-out test set (**Supplementary Table 2**).

# Supplementary Figure 1


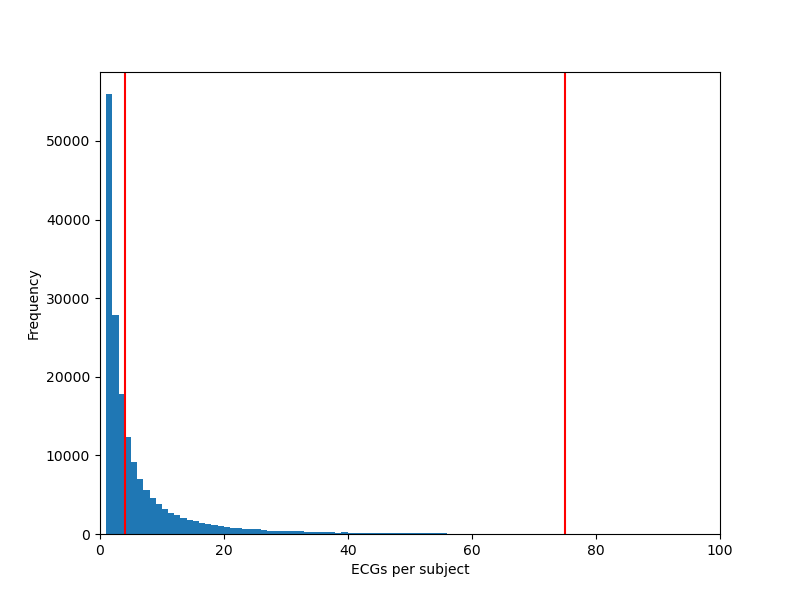


**Distribution of ECGs per subject in the whole BIDMC cohort.** Red lines denote cutoff of 4 to 75 ECGs per subject, which is the range included in our study. *BIDMC, Beth Israel Deaconess Medical Center.*

# Supplementary Figure 2


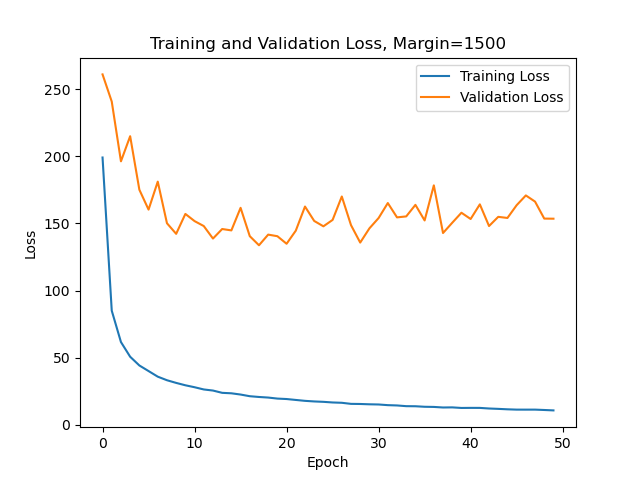
**a**


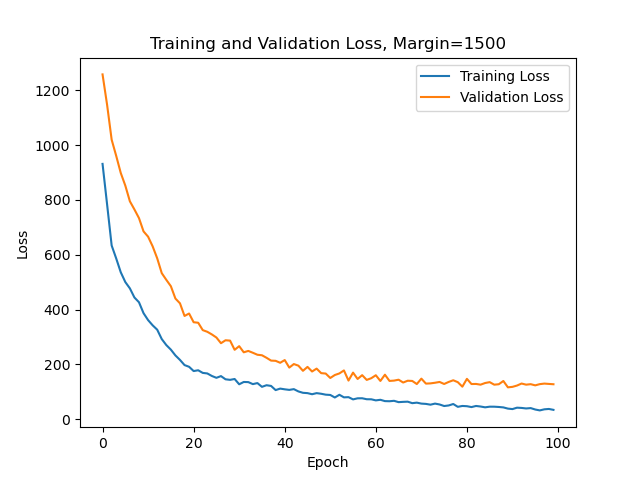
**b**

**Training and validation loss curves. (a)** SNN-ECG, **(b)** SNN-ECG-Normal. Both panels show a falling training and validation loss with each training epoch, until a validation loss plateau is reached. The validation loss plateau is reached in fewer epochs in SNN-ECG as compared to SNN-ECG-Normal due to the much larger sample size for SNN-ECG. *SNN, Siamese neural network.*

# Supplementary Figure 3

**
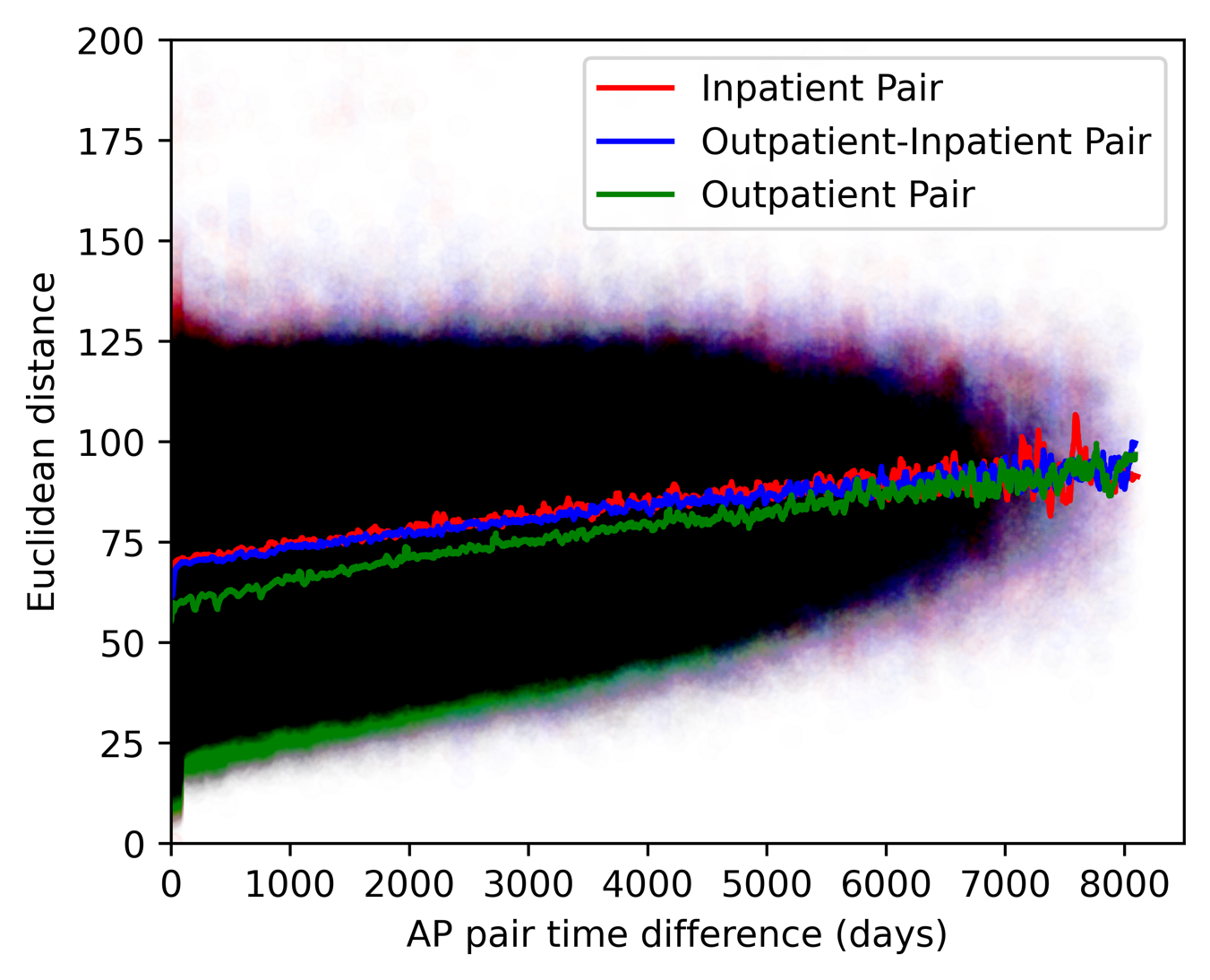
**

**The effect of time between AP (i.e. same subject) ECG acquisition and the AP Euclidean distance as output by SNN-ECG**. The Euclidean distance increases with the time between ECG acquisition. The 30-day rolling average Euclidean distance for AP pairs comprising both outpatient ECGs (green), both inpatient ECGs (red) and an outpatient and inpatient ECG (blue) shows the higher similarity (i.e. shorter Euclidean distance) between two outpatient ECGs from the same subject diminishes over the time-interval range compared to inpatient and outpatient-inpatient pairs. *AP, anchor-positive; SNN, Siamese neural network.*

# Supplementary Table 1

|  | **Binary threshold** |
| --- | --- |
| **All** | 97.767 |
| **Outpatient** | 96.092 |
| **Normal** | 93.366 |
| **LBBB** | 77.076 |
| **RBBB** | 83.097 |
| **AF** | 87.214 |

**Binary thresholds between AP and AN pairs.** *AF, atrial fibrillation; AN, anchor-negative; AP, anchor-positive; LBBB, left bundle branch block; RBBB, right bundle branch block.*

# Supplementary Table 2

|  | **Total triplets** | **Accuracy (%)** | **Sensitivity** | **Specificity** | **PPV** | **NPV** |
| --- | --- | --- | --- | --- | --- | --- |
| **All** | 2,689,124 | 82.16 | 0.758 | 0.885 | 0.869 | 0.785 |
| **Outpatient** | 686,844 | 85.62 | 0.797 | 0.915 | 0.904 | 0.819 |
| **Normal** | 7,600 | 91.29 | 0.904 | 0.922 | 0.920 | 0.906 |
| **LBBB** | 69,962 | 74.64 | 0.710 | 0.783 | 0.766 | 0.730 |
| **RBBB** | 134,872 | 76.83 | 0.666 | 0.871 | 0.837 | 0.723 |
| **AF** | 142,147 | 82.16 | 0.778 | 0.865 | 0.852 | 0.796 |

**SNN-ECG-Normal results for the hold-out test set and ECG subsets.** *AF, atrial fibrillation; LBBB, left bundle branch block; NPV, negative predictive value; PPV, positive predictive value; RBBB, right bundle branch block; SNN, Siamese neural network.*

# Supplementary Table 3

|  | **n subjects with ≥ 2 ECGs** |
| --- | --- |
| **All** | 21,737 |
| **Outpatient** | 14,381 |
| **Normal** | 1,539 |
| **LBBB** | 1,141 |
| **RBBB** | 1,777 |
| **AF** | 3,093 |

**Subjects with at least 2 ECGs in the hold-out test set.** *AF, atrial fibrillation; LBBB, left bundle branch block; RBBB, right bundle branch block.*

# Supplementary Table 4

|  | **Dataset size** | **Top n choice(s)** | **No supplemental information (%)** | **Sex (%)** | **Decade age bracket (%)** | **Sex and decade age bracket (%)** |
| --- | --- | --- | --- | --- | --- | --- |
| **All** | **100** | 1 | 79.2 | 82.8 | 88.9 | 91.4 |
|  |  | 2 | 86.1 | 89.2 | 94.0 | 96.0 |
|  |  | 5 | 92.0 | 94.5 | 97.6 | 99.0 |
|  |  | 10 | 95.3 | 97.0 | 99.3 | 99.9 |
|  | **1,000** | 1 | 62.6 | 66.4 | 74.7 | 78.4 |
|  |  | 2 | 70.3 | 74.2 | 81.8 | 85.2 |
|  |  | 5 | 78.5 | 82.1 | 88.6 | 91.3 |
|  |  | 10 | 83.4 | 86.7 | 92.5 | 94.8 |
|  | **10,000** | 1 | 45.0 | 48.2 | 56.9 | 60.6 |
|  |  | 2 | 52.2 | 56.0 | 64.4 | 68.5 |
|  |  | 5 | 60.6 | 64.3 | 73.3 | 77.3 |
|  |  | 10 | 66.4 | 70.6 | 79.0 | 82.4 |
|  | **20,000** | 1 | 40.0 | 43.1 | 51.3 | 54.7 |
|  |  | 2 | 46.7 | 50.1 | 58.9 | 62.6 |
|  |  | 5 | 55.0 | 58.5 | 67.5 | 71.4 |
|  |  | 10 | 60.8 | 64.6 | 73.4 | 77.3 |
| **Outpatient** | **100** | 1 | 85.3 | 87.7 | 92.2 | 94.0 |
|  |  | 2 | 90.3 | 92.4 | 95.6 | 97.0 |
|  |  | 5 | 94.8 | 96.3 | 98.4 | 99.2 |
|  |  | 10 | 96.7 | 98.0 | 99.5 | 99.9 |
|  | **1,000** | 1 | 70.8 | 73.8 | 80.6 | 83.4 |
|  |  | 2 | 77.7 | 80.8 | 86.9 | 89.0 |
|  |  | 5 | 84.6 | 87.3 | 91.7 | 93.6 |
|  |  | 10 | 88.6 | 90.9 | 94.4 | 96.0 |
| **Normal** | **100** | 1 | 89.4 | 91.4 | 95.1 | 96.3 |
|  |  | 2 | 94.0 | 95.8 | 98.0 | 98.9 |
|  |  | 5 | 97.8 | 98.7 | 99.5 | 99.7 |
|  |  | 10 | 98.8 | 99.3 | 99.7 | 100.0 |
|  | **1,000** | 1 | 72.5 | 75.9 | 84.2 | 87.5 |
|  |  | 2 | 80.1 | 84.3 | 91.6 | 93.7 |
|  |  | 5 | 88.0 | 90.8 | 95.8 | 97.1 |
|  |  | 10 | 93.0 | 94.9 | 97.4 | 98.6 |
| **LBBB** | **100** | 1 | 64.6 | 70.1 | 77.7 | 82.5 |
|  |  | 2 | 73.1 | 79.1 | 86.2 | 90.1 |
|  |  | 5 | 84.1 | 89.1 | 93.9 | 97.3 |
|  |  | 10 | 89.7 | 93.3 | 97.5 | 99.5 |
|  | **1,000** | 1 | 44.6 | 49.1 | 57.7 | 62.6 |
|  |  | 2 | 52.2 | 57.3 | 66.3 | 72.7 |
|  |  | 5 | 62.1 | 68.4 | 76.5 | 81.7 |
|  |  | 10 | 69.6 | 75.4 | 84.0 | 88.3 |
| **RBBB** | **100** | 1 | 66.7 | 71.9 | 77.8 | 81.6 |
|  |  | 2 | 74.2 | 79.1 | 85.6 | 89.3 |
|  |  | 5 | 83.2 | 87.6 | 93.5 | 96.1 |
|  |  | 10 | 88.0 | 92.2 | 97.1 | 98.6 |
|  | **1,000** | 1 | 51.9 | 55.2 | 62.5 | 66.3 |
|  |  | 2 | 57.3 | 61.5 | 69.3 | 73.5 |
|  |  | 5 | 65.8 | 69.6 | 77.0 | 81.6 |
|  |  | 10 | 71.7 | 76.1 | 82.6 | 87.2 |
| **AF** | **100** | 1 | 73.0 | 77.2 | 83.7 | 87.7 |
|  |  | 2 | 80.7 | 85.6 | 90.5 | 93.3 |
|  |  | 5 | 88.3 | 91.5 | 95.7 | 97.6 |
|  |  | 10 | 92.5 | 95.5 | 98.0 | 99.5 |
|  | **1,000** | 1 | 54.2 | 58.6 | 67.2 | 71.0 |
|  |  | 2 | 62.6 | 66.8 | 75.2 | 79.2 |
|  |  | 5 | 71.3 | 75.8 | 82.6 | 86.3 |
|  |  | 10 | 77.2 | 81.9 | 87.9 | 91.3 |

**SNN-ECG success rates for subject re-identification from anonymised datasets.** *AF, atrial fibrillation; LBBB, left bundle branch block; RBBB, right bundle branch block; SNN, Siamese neural network.*

# Supplementary Table 5

|  | **95% cut-off in dataset of 100** | **95% cut-off in dataset of 1,000** |
| --- | --- | --- |
| **All** | 17.9986 | 17.3731 |
| **Outpatient** | 17.8679 | 17.9836 |
| **Normal** | 13.7316 | 17.0902 |
| **LBBB** | 20.7732 | 15.0396* |
| **RBBB** | 17.1091 | 15.3634^†^ |
| **AF** | 22.0923 | 31.4007 |

**Cut-offs at 95% certainty.** * only 516 LBBB subjects in the validation set. ^†^ only 674 RBBB subjects in the validation set. *AF, atrial fibrillation; LBBB, left bundle branch block; RBBB, right bundle branch block.*

# References to Supplementary Appendix

1. Sau A, Pastika L, Sieliwonczyk E, Patlatzoglou K, Ribeiro AH, McGurk KA, et al. Artificial intelligence-enabled electrocardiogram for mortality and cardiovascular risk estimation: a model development and validation study. Lancet Digit Health. 2024 Nov;6(11):e791–802.

2. Pastika L, Sau A, Patlatzoglou K, Sieliwonczyk E, Ribeiro AH, McGurk KA, et al. Artificial intelligence-enhanced electrocardiography derived body mass index as a predictor of future cardiometabolic disease. NPJ Digit Med. 2024 Jun 25;7(1):167.

3. Schroff F, Kalenichenko D, Philbin J. FaceNet: A unified embedding for face recognition and clustering. In: 2015 IEEE Conference on Computer Vision and Pattern Recognition (CVPR). IEEE; 2015. p. 815–23.
